# The Effect of Complex Modifier Consisting of Star Macromolecules and Ionic Liquid on Structure and Gas Separation of Polyamide Membrane

**DOI:** 10.3390/membranes13050516

**Published:** 2023-05-15

**Authors:** Ilya Faykov, Galina Polotskaya, Ivan Kuryndin, Zoolsho Zoolshoev, Natalia Saprykina, Nadezhda Tian, Angelina Sorokina, Alexandra Pulyalina

**Affiliations:** 1 Institute of Chemistry, Saint Petersburg State University, 198504 Saint Petersburg, Russia; st022544@student.spbu.ru (I.F.); polotskaya@hq.macro.ru (G.P.); tyan-nadezhda91@yandex.ru (N.T.); gilllya@mail.ru (A.S.); 2 Institute of Macromolecular Compounds, Russian Academy of Sciences, 199004 Saint Petersburg, Russia; isk76@mail.ru (I.K.); zoolfar@mail.ru (Z.Z.); saprykina@hq.macro.ru (N.S.)

**Keywords:** poly(*m*-phenylene isophthalamide), membrane, complex modifier, gas separation

## Abstract

A novel hybrid membrane was developed on the basis of poly(*m*-phenylene isophthalamide) (PA) by introducing an original complex modifier into the polymer; this modifier consisted of equal amounts of heteroarm star macromolecules with a fullerene C_60_ core (HSM) and the ionic liquid [BMIM][Tf_2_N] (IL). The effect of the (HSM:IL) complex modifier on characteristics of the PA membrane was evaluated using physical, mechanical, thermal, and gas separation techniques. The structure of the PA/(HSM:IL) membrane was studied by scanning electron microscopy (SEM). Gas transport properties were determined by measuring He, O_2_, N_2_, and CO_2_ permeation through the membranes based on PA and its composites containing a 5 wt% modifier. The permeability coefficients of all gases through the hybrid membranes were lower than the corresponding parameters for the unmodified membrane, whereas the ideal selectivity in the separation of He/N_2_, CO_2_/N_2,_ and O_2_/N_2_ gas pairs was higher for the hybrid membrane. The position of the PA/(HSM:IL) membrane on the Robeson’s diagram for the O_2_/N_2_ gas pair is discussed.

## 1. Introduction

Energy and ecological crises that permanently exist in modern industry necessitate the search for ways to decrease energy consumption and reduce environmental pollution. In recent years, membrane technologies, including polymer electrolyte fuel cells, reverse osmosis, dialysis, etc., have been successfully applied to solve these important problems, because membrane processes possess such advantages as environmental friendliness, low power consumption, compact equipment, and high performance [1,2]. Membrane gas separation is currently being actively developed and widely used in chemical and petrochemical plants for the separation of hydrogen from various streams, removal of carbon dioxide from natural gas, separation of nitrogen from air, etc. [3,4,5,6,7]. However, to expand the scope of existing applications of membrane technologies, it is necessary to develop membrane materials with improved transport properties [8,9,10]. The modification of well-established membrane polymers makes it possible to change the original properties of the polymers and impart new characteristics to the materials [11,12,13].

It has been shown that the use of various inorganic particles, including carbon nanoparticles, metal–organic frameworks, and silica as modifiers can significantly enhance membrane transport characteristics [14,15,16]. However, in the process of forming hybrid membranes, various defects often occur within the membrane structure due to poor compatibility between nanoparticles and polymers, and the presence of these defects reduces the efficiency of a separation process [17,18]. Over the last years, the use of modified particles with a functionalized surface has proven to be a promising approach to improving membrane characteristics without destructing a polymer matrix [19].

In our previous works, fullerene C_60_ molecules have been modified by anionic polymerization and served as branching centers of stars with twelve macromolecular arms [20,21,22]. The star-shaped modifier has been used as filler in polymer membranes, which have acquired enhanced transport properties in pervaporation and gas separation. The star modifiers exhibited a good compatibility with the membrane matrix of poly(*m*-phenylene isophthalamide) (PA) or poly(phenylene oxide) (PPO) and dramatically changed the internal structure of the membranes by forming its cellular morphology and building extended nanoscale and submicron channels in the membrane structure [21,23].

Currently, ionic liquids (ILs) are extensively used to modify membrane polymers [24,25,26,27,28]. It is known that ILs possess a number of unique properties, such as low vapor pressure, non-volatility, non-flammability, and high ionic conductivity; moreover, they are considered to be environmentally friendly [26,29]. It has been established that the gas transport properties of polymeric membranes were improved upon the introduction of ILs [30,31].

This work is focused on the complex modifier consisting of equal amounts of heteroarm star-shaped macromolecules (HSM) and 1-butyl-3-methylimidazolium bis(trifluoromethylsulfonyl)imide [BMIM^+^Tf_2_N^−^] as an IL. HSM is a twelve-arm star with six arms of polystyrene (PS) and six arms of diblock copolymer poly-2-vinylpyridine-*block*-poly-*tert*-butylmethacrylate (P2VP-*block*-PTBMA) that are covalently attached to a fullerene (C_60_) branching center [32]. This complex filler has been used as a modifier of the PPO matrix in the work [33] devoted to studies of dehydration of lactic acid by pervaporation. It has been found that a special compatibility between the PPO polymer matrix and PS arms of star-shaped macromolecules led to an enhanced total flux and separation factor of the prepared membranes [34].

In this work, commercial poly(*m*-phenylene isophthalamide) (PA) was selected as a polymer matrix. This polymer is a commonly used material for diffusion membranes [35,36,37,38,39,40] since it exhibits outstanding mechanical performance and high thermal stability due to the formation of a hydrogen bond network.

The aim of this work included the development of a new hybrid PA-based membrane, filled with the complex modifier consisting of equal amounts of HSM and IL. We assume that possible interactions between the PA matrix and [BMIM^+^Tf_2_N^−^] can occur by means of hydrogen bonding, similar to what was found in the work [41]. The experimental study of hybrid membrane characteristics and a detailed analysis of the changes in the structure and properties of the PA membrane caused by the introduction of 5 wt% of the (HSM:IL) complex modifier were the goals of this present work. Furthermore, special attention was given to the investigation of the effect of the (HSM:IL) complex modifier on the gas separation properties of the hybrid membrane.

## 2. Materials and Methods

### 2.1. Materials

**Figure 1:**
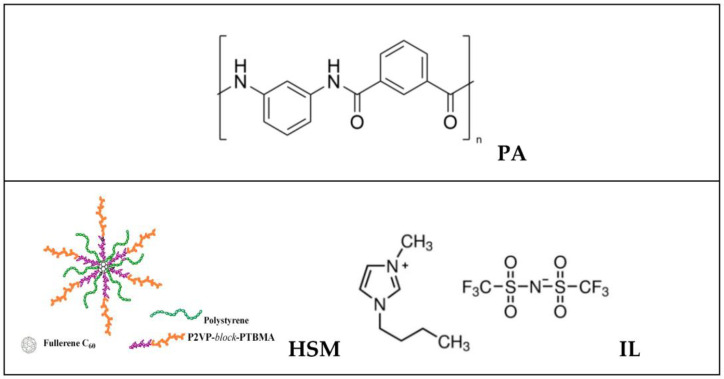
Schematic representation of components: PA, heteroarm star macromolecule (HSM), and ionic liquid [BMIM][Tf_2_N] (IL).

Poly(*m*-phenylene isophthalamide) (PA, brand name Phenylon^®^, Uniplast, Vladimir, Russia) was used as a basic membrane material. Figure 1 shows the structures of the components used to prepare the hybrid membrane. Heteroarm star macromolecules (HSM) were obtained by multistage synthesis that involved the attachment of two types of polymer chains to a fullerene (C_60_) branching center by anionic process [42]. The resultant macromolecule consisted of six arms of nonpolar polystyrene (PS) and six arms of polar diblock copolymer poly-2-vinylpyridine-*block*-poly-*tert*-butylmethacrylate (P2VP–*block*-PTBMA) grafted onto a C_60_ core. The molecular weight of the PS arm was *M_n_* = 6900 g/mol (*M_w_/M_n_* = 1.04), based on the data of size-exclusion chromatography. According to the synthesis conditions, the molecular weight of the diblock copolymer arm was two times higher than that of the PS arm, whereas the P2VP and PTBMA blocks had equal lengths.

Ionic liquid (IL) 1-butyl-3-methylimidazolium bis(trifluoromethylsulfonyl)imide [BMIM][Tf_2_N] was purchased from Sigma–Aldrich Chemie GmbH (Schnelldorf, Germany). *N*,*N*-Dimethylacetamide (DMA) manufactured by Vekton (Saint Petersburg, Russia) was used as a solvent, without further purification.

### 2.2. Membrane Preparation

The (HSM:IL) complex modifier was prepared by mixing the components in a 1:1 ratio (wt%) in DMA solution at a concentration of 4 wt%. The resulting solution was kept under steady-state conditions for 24 h, so that the interactions between the HSM and IL molecules could take place. After this, the modifier solution was sonicated for 40 min.

A casting solution of PA/(HSM:IL) in a ratio of 95/5 was prepared by mixing an 8 wt% PA solution and 4 wt% (HSM:IL) solution in DMA, in calculated amounts. The mixture was intensively stirred for one hour and left for three days to ensure a complete interaction between the PA and (HSM:IL). Following this, the solution was sonicated for 40 min and filtered. The PA/HSM casting solution in the 95/5 ratio was prepared in a similar way.

Dense membranes based on PA and its composites (PA/HSM or PA/(HSM:IL)) containing 5 wt% filler were obtained by casting a 2 wt% polymeric solution in DMA on a cellophane surface and placed on a balanced table in a thermostat. The solvent was removed by evaporation at 60 °C, and the membrane was separated from the cellophane substrate and dried in a vacuum oven at 60 °C until a constant weight was reached. The membrane thickness was ~30 µm.

### 2.3. Characterization

Membrane morphology was studied using a Zeiss SUPRA 55VP scanning electron microscope (Carl Zeiss, Oberkochen, Germany) equipped with In-lens SE and SE2 secondary electron detectors, a secondary electron detector for low-vacuum mode (VPSE), and a 4-quadrant backscattered electron detector (AsB). The dried membrane samples were cracked in liquid nitrogen and then sputtered with a 20 nm thick platinum layer using the Quorum 150 cathode sputtering installation (Quorum Technologies Ltd., Lewes, UK) before the experiment.

The presence of functional groups was investigated by FTIR spectroscopy with the aid of a Bruker Tensor 27 FTIR spectrometer (Bruker Daltonics, Bremen, Germany), with a resolution of 1 cm^−1^ in the range 4000–500 cm^−1^ at 25 °C.

Thermogravimetric analysis (TGA) was performed on the TG 209 F3 Iris thermo-microbalance (Netzsch, Selb, Germany) using samples of ∼10 mg in the process of a dynamic temperature increase (from 25 to 500 °C) in an inert atmosphere with a nitrogen flow of 50 cm^3^/min.

The glass transition temperature (*T_g_*) was determined by thermomechanical analysis, using a TMA402 F1 Hyperion apparatus (NETZSCH, Germany) at a heating rate of 5 °C/min.

The membrane density (*ρ*) was estimated using the flotation method with a laboratory-made measurement unit. The mixture of toluene and carbon tetrachloride was used to equilibrate the specimens at 20 °C (*ρ_Toluene_* = 0.867 g/cm^3^, *ρ_CCl_*_4_ = 1.594 g/cm^3^). The weight of used samples was 0.05–0.10 g; the error of measurements was ±0.0001 g/cm^3^. The specific volume (*V*_0_) of a polymer membrane was determined as *V*_0_ *=* 1/*ρ*.

Intermolecular interactions between PA, HSM, and (HMS:IL) in DMA were studied with capillary viscometry. The relative and intrinsic viscosities of pristine PA and its mixtures with HSM or (HSM:IL) in DMA solutions were measured using an Ubbelohde viscometer at 20 °C. The analyzed solutions of polymer mixtures of PA and HSM or PA and (HSM:IL) in the entire range of component concentrations (0–100 wt%) were prepared by mixing 0.5 wt% solutions of PA and the modifier in DMA. Relative viscosity (*η_rel_*) was calculated by the equation [43]:

*η_rel_* = *η_c_*/*η*_0_, (1)

where *η_c_* is the viscosity of the polymer mixture solution, and *η*_0_ is the viscosity of a pure solvent. In practice, the *η_c_*/*η*_0_ ratio is defined as the expiration time of equal volumes of the polymer blend solution and pure solvent through the same calibrated capillary. The error of the experiment did not exceed 2%.

The additive relative viscosity (*η_rel_*)*_add_* was calculated as the sum of the fractions of the relative viscosities of the mixture components:

(*η_rel_*)*_add_* = *η*_*rel*1_ (*w*_1_) + *η*_*rel*2_ (*w*_2_), (2)

where *η_rel_* is the relative viscosity of each component of the mixture (1—PA, 2—HSM or (HSM:IL)), and *w* is their volume fraction in the solution.

To determine the tensile strength, yield strength, elastic modulus, and elongation at break, mechanical tests of membranes were carried out using a 2166 R-5 tensile test machine (Tochpribor, Ivanovo, Russia). Specimens with a size of 5 × 50 mm were fixed in vices-type clamps, and stress–strain curves were recorded at uniaxial extension with a crosshead rate of 50 mm/min. For each specimen, the test was repeated at least five times.

### 2.4. Gas Separation Tests

The gas permeability parameters of the membranes were measured using high-purity single gases (He, O_2_, N_2_, and CO_2_). Table 1 lists the effective molecular diameters for these gases, derived from the statistical treatment of gas permeability data of polymers and statistical parameters [44]. The experiments were carried out according to the barometric technique, with a laboratory high-vacuum apparatus that included a static permeation cell with an effective area of 5.25 cm^2^ at 30 °C. A membrane sample was placed into a module, which was sealed and evacuated. At the beginning of the permeation experiment, the tested gas was brought into the feed part of the permeation cell under a constant pressure *p* (150 kPa). The permeability was determined from a pressure increase Δ*p* in a calibrated volume *V_p_* of the product part of the cell per the time interval Δ*t* during steady-state permeation. The gas permeability coefficient (*P*) was calculated according to the following equation:

(3) $$P=\frac{\Delta p}{\Delta t}\cdot \frac{V_{p}\cdot l}{S\cdot p}\cdot \frac{1}{RT},$$

where *l* is the membrane thickness, *S* is the membrane area, *T* is the absolute temperature, and *R* is the gas constant. The permeability coefficient *P* was expressed in Barrers (1 Barrer = 10^−10^ cm^3^ (STP) cm/(cm^2^ s cmHg)). Each experiment was repeated 3 times to ensure the data reproducibility, and several membrane samples having approximately the same thickness and prepared under the same conditions were used.

The ideal selectivity (*α*_*i*/*j*_) for gas *i* with respect to gas *j* was calculated, according to the following equation:

(4) $$\alpha_{i/j}=\frac{P_{i}}{P_{j}},$$

## 3. Results

### 3.1. Physical Properties

The effect of the (HSM:IL) complex modifier on the structure and properties of PA-based membranes was estimated during the comparative study of pure PA, PA/HSM, and PA/(HSM:IL) samples containing a 5 wt% modifier.

As noted above, the first step in the preparation of composite (hybrid) PA/HSM and PA/(HSM:IL) membranes consists in mixing solutions of the matrix polymer and components of the modifier in DMA. In multicomponent systems, specific intermolecular polymer–polymer and polymer–solvent interactions can occur at this stage. These interactions lead to a change in the size of macromolecular coils and, as a consequence, to a change in the viscosity of the polymer system [45,46,47]. Capillary viscometry was used to study the intermolecular interactions of PA and HSM and PA/(HSM:IL) systems in DMA.

**Figure 2:**
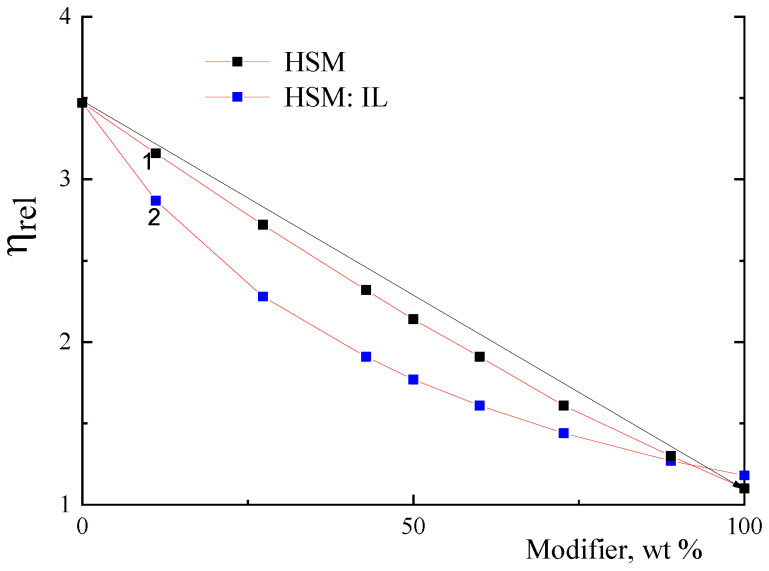
Dependences of the relative viscosity (*η_rel_*) of (1) PA/HSM and (2) PA/(HSM:IL) samples in DMA on the content of an HSM or (HSM:IL) modifier at 20 °C.

Figure 2 shows the relative viscosity (*η_rel_*) of the PA and HSM mixture (curve 1) and PA and (HSM:IL) (curve 2) in the DMA solutions as the dependence on the modifier content. The straight line in Figure 2 represents the additive relative viscosity (*η_rel_*)*_add_* calculated (by Equation (2)) in the absence of any interactions between the polymer components of the mixture in dilute solutions.

It is seen that curves 1 and 2 representing the experimentally obtained values of *η_rel_* for PA/HSM and PA/(HSM:IL) solutions have a noticeable negative deviation from the straight line. Whilst the deviation of curve 1 for the PA and HSM system from the additive value (*η_rel_*)*_add_* is only 4% (a very weak interaction), for the PA system with (HSM:IL) additives this deviation reaches 20%.

A negative deviation from the additive value (*η_rel_*)*_add_* and/or a decrease in relative viscosity when adding a second component to the system indicates that the hydrodynamic volume of macromolecules in the mixture is less than the sum of volumes of the mixture components in the absence of interaction between them. Thus, the use of the complex modifier HSM:IL leads to an even greater deviation of *η_rel_* from the additive value (*η_rel_*)*_add_*, since the macromolecules become more compact.

The intrinsic viscosity of the PA sample decreases after adding HSM and (HSM:IL) (Table 2), indicating that the PA macromolecules acquire more compact conformation. This result is consistent with the data of capillary viscometry (Figure 2), which are suggestive of the existence of an intermolecular interaction between membrane components in the solution. A decrease in viscosity of the system is accompanied by a regular decrease in the glass transition temperature. As can be seen from Table 2, membrane density decreases after modifying the PA membrane with HSM and (HSM:IL). One can reasonably suggest that the complex supramolecular structures appearing in the solution form membranes with a lower packing density during drying, i.e., the evaporation of the solvent.

**Figure 3:**
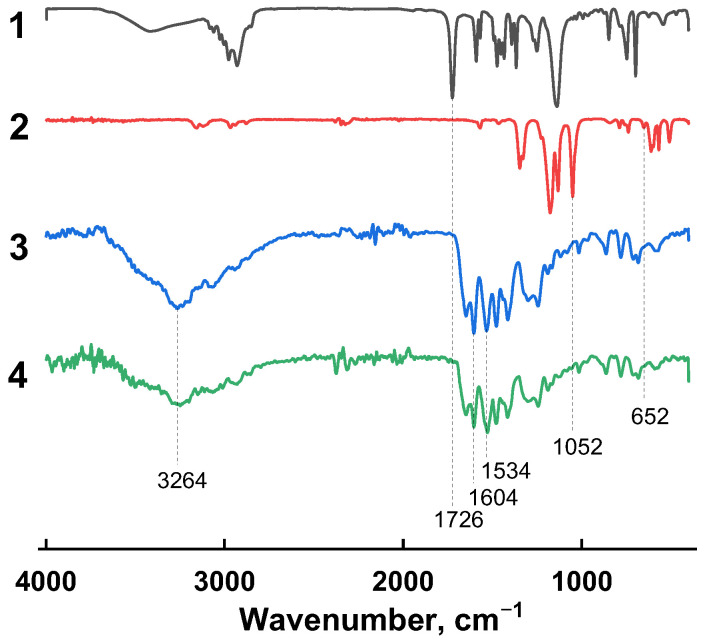
FTIR spectra of (1) HSM, (2) IL, (3) PA, and (4) PA/(HSM:IL).

Further, the membranes under study were analyzed by FTIR to prove (or disprove) the existence of chemical interactions between the additives and the matrix polymer. Figure 3 shows the FTIR spectra of the PA and PA/(HSM:IL) membranes, as well as individual HSM and IL substances. The characteristic bands appeared in the HSM spectra in the region of 1726 cm^−1^ are the C=O stretching vibrations of the ester group. The characteristic absorption band for IL appeared at 1052 cm^−1^, due to stretching vibrations of the C–N bond in the N-heterocyclic ring of the IL cation. The peaks observed at 652 cm^−1^ are associated with bending vibrations of the S–N–S bond of the IL anion. The peaks at 1534 cm^−1^ and 1604 cm^−1^ in the PA spectra are associated with N–H bending and C=O stretching in the amide bond. The peak at 3264 cm^−1^ has a lower intensity in the spectra of hybrid membranes, which can be caused by the disappearance of a large number of hydrogen bonds as a result of the release of N–H in PA. Furthermore, no new groups are formed in the structure of the hybrid membranes. One may conclude that the combination of a PA and (HSM:IL) complex modifier is achieved through physical interactions rather than chemical ones. Therefore, the results of FTIR spectroscopy are indicative of a successful modification of the membrane with the complex modifier.

The stability of IL as a component of the complex modifier (HSM:IL) in the polymer membrane has been shown in our previous works [33,34], where data of the quantum chemical calculations demonstrated strong coordination between IL and the HSM, which occurs mainly due to the interaction of the IL cation (BMIM^+^) and diblock copolymer (P2VP-*block*-PTBMA). Furthermore, the FTIR spectra obtained for the hybrid membranes supported the presence of IL in the membrane structure both before and after separation tests. Therefore, it can be concluded there is no loss of IL during mass transfer through the membranes.

### 3.2. Membrane Structure

**Figure 4:**
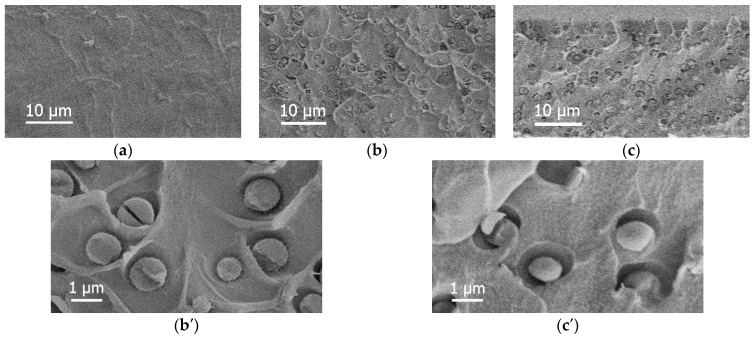
SEM images of cross-sections for (**a**) PA, (**b**,**b′**) PA/HSM, and (**c**,**c′**) PA/(HSM:IL) membranes.

The membrane morphology was studied by SEM. Figure 4 shows the SEM micrographs of cross-sections of the PA, PA/HSM, and PA/(HSM:IL) membranes. According to the presented micrographs, the cross-section of the unmodified PA membrane is rather dense and uniform. In the hybrid membranes, structuring occurs owing to the presence of HSM particles, which form spherical domains 0.3–2 μm in size and are uniformly distributed in the PA matrix. The domains are formed due to the segregation of the polar arms of the HSM. The boundaries of the cellular structure are somewhat smoothed in the membrane comprising the complex modifier (HSM:IL); probably, IL and HSM are concentrated therein.

### 3.3. Thermal Stability

**Figure 5:**
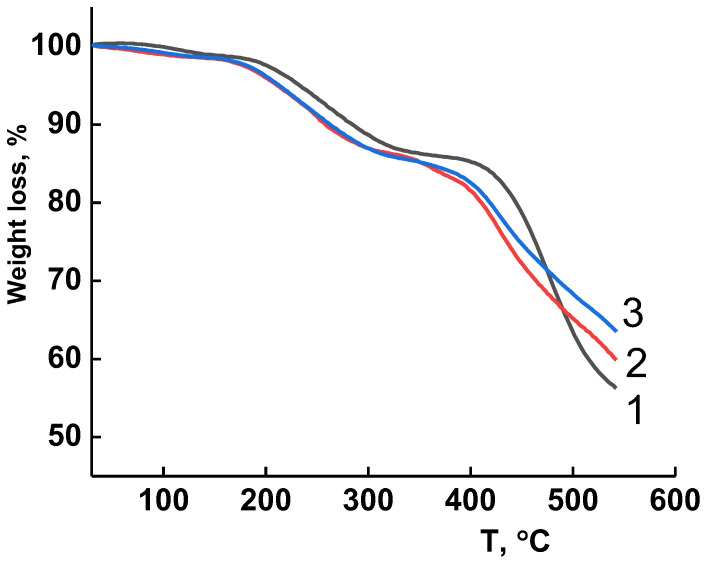
TG curves of membranes (1) PA, (2) PA/(HSM:IL), and (3) PA/HSM.

The results of studying the thermal stability of the membranes by TGA are shown in Figure 5. In general, no differences in the trends of weight loss are observed for all the membranes studied. In the temperature range up to 165 °C, a slight weight loss of approximately 2 wt% is registered, which can be caused by a release of moisture and low molecular weight impurities absorbed on the membrane surface. In the range from 165 °C to 380 °C, the weight loss is induced by the removal of the residual solvent (DMA). Significant weight loss for all the membranes under study begins at temperatures above 385 °C and is related to thermal degradation processes. Therefore, the prepared membranes have high thermal stability and can operate in a wide temperature range.

### 3.4. Gas Transport Properties

**Figure 6:**
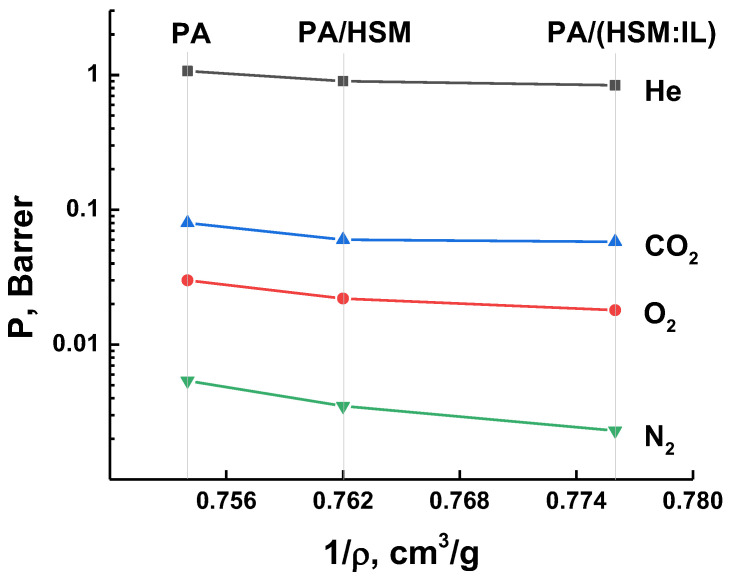
Permeability coefficients of He, O_2_, N_2_, and CO_2_ as a function of the specific volume (1/*ρ*) of PA, PA/HSM, and PA/(HMS:IL) membranes.

The gas transport properties of the PA, PA/HSM, and PA/(HSM:IL) membranes were estimated using the data on the permeation of the single permanent gases He, O_2_, N_2_, and CO_2_. Figure 6 demonstrates the data on the permeability coefficients of He, O_2_, N_2_, and CO_2_ for the studied membranes. The level of gas permeability is determined by the effective kinetic diameter of gas molecules (Table 1). Therefore, gas permeability values decrease with increasing the molecular diameter of gases in the following order: He > CO_2_ > O_2_ > N_2_. Furthermore, it can be seen that gas permeation through membranes is proportional to the specific volume of the membrane, which decreases after the introduction of the modifier (Table 3). This may be due to the fact that the presence of the modifier leads to the compactization of macromolecules and formation of aggregates in the casting solution. At the same time, upon solvent evaporation, the segregation of matrix and filler phases occurs. As a result, the size distribution and topology of the free volume elements in the membranes undergo certain changes, which manifest themselves in a slight decrease in the permeability of the PA/(HSM:IL) hybrid membrane.

**Figure 7:**
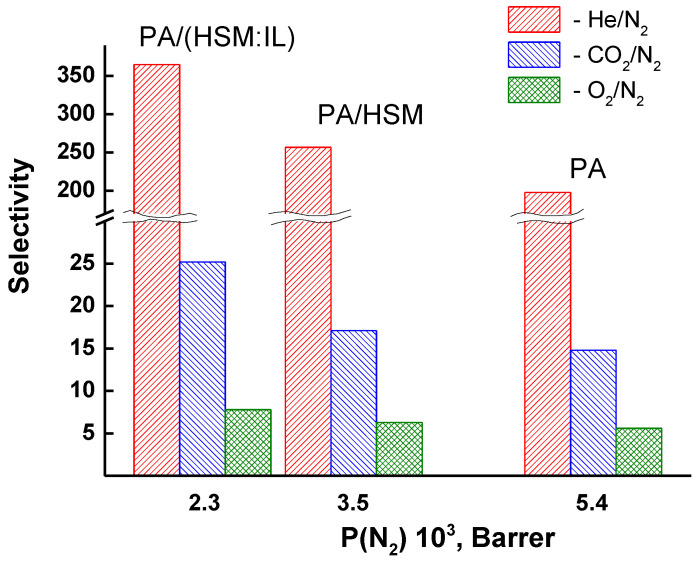
Dependence of ideal selectivity in separation of He/N_2_, CO_2_/N_2_, and O_2_/N_2_ gas pairs on nitrogen permeability using PA, PA/HSM, and PA/(HMS:IL) membranes.

Figure 7 shows the dependence of ideal selectivity in the separation of He/N_2_, CO_2_/N_2_, and O_2_/N_2_ gas pairs using the PA, PA/HSM, and PA/(HMS:IL) membranes. Separation capacity is improved upon inclusion of a 5 wt% HSM or (HMS:IL) modifier into the PA matrix. This result can be explained by a change in the internal structure and redistribution of the free volume in the hybrid membranes.

**Figure 8:**
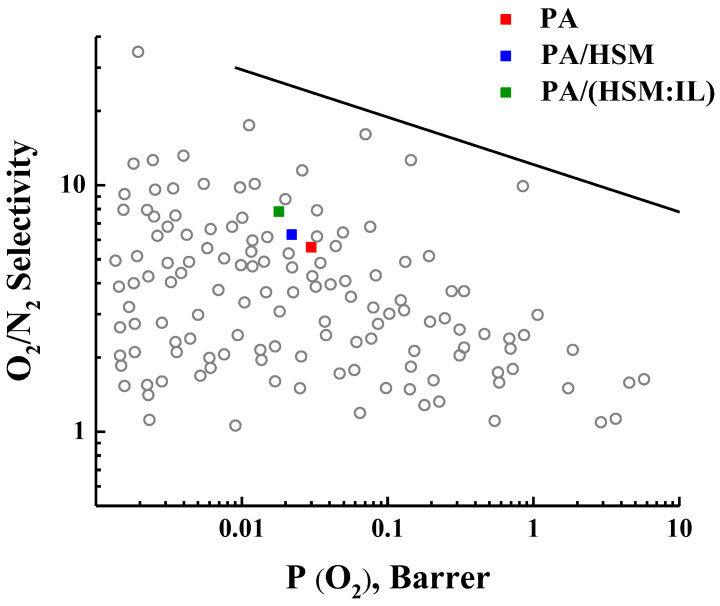
O_2_/N_2_ selectivity as a function of oxygen permeability. The upper bound line was taken from Robeson’s diagrams [50].

To assess the separation efficiency of the studied PA-based membranes, their transport properties were plotted on Robeson’s diagram [48]. L.M. Robeson analyzed data on permeability and selectivity for a large number of polymers and established the position of the upper bound line for several gas pairs. Said upper bound line corresponds to the high level of selectivity vs. permeability for membranes known from the literature up to 1991 [49] and later up to 2008 [50]. Figure 8 shows the upper bound lines for the membrane separation of the O_2_/N_2_ mixture [50] and data on O_2_ permeability and O_2_/N_2_ selectivity for the PA, PA/HSM, and PA/(HMS:IL) membranes. It is evident that our membranes exhibit reasonable properties, and the position of the PA membrane is improved as it moves to higher O_2_/N_2_ selectivity upon introducing HMS and especially (HSM:IL) into the PA matrix. This result demonstrates that the use of our complex fillers is a promising way of membrane modification.

### 3.5. Mechanical Properties

**Figure 9:**
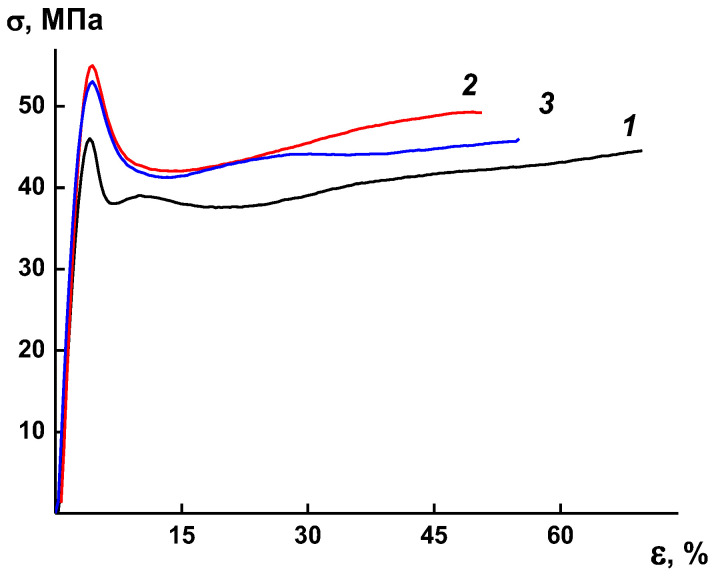
Stress–strain curves (σ–ε) of membranes PA (1), PA/HSM (2), and и PA/(HSM:IL) (3).

To establish the effect of the complex modifier (HSM:IL) on the deformation properties of the membranes, the mechanical properties of PA, PA/HSM, and PA/(HMS:IL) films were analyzed. As can be seen from Figure 9, the stress–strain curves of the films are typical of polymers. In the initial region, the samples demonstrate elastic deformation characterized by the elastic modulus (Young’s modulus). Furthermore, as the samples are strained, a clearly defined yield point is observed followed by a range of plastic deformation.

According to the obtained stress–strain curves, the mechanical characteristics of the samples were calculated (Table 3). As seen from the presented data, the addition of 5% HSM leads to an increase in strength, yield strength, and elastic modulus of the composite as compared to those of the unmodified PA membrane. At the same time, a decrease in the elongation at break is observed, although it remains relatively high. In terms of deformation behavior, the PA/(HSM:IL) composite system occupies an intermediate position between the PA/HSM sample and unmodified PA membrane.

Thus, mechanical tests of the PA films and PA-based composite systems revealed a high strength and elongation at break, which will ensure the processability of the materials required for their practical applications.

## 4. Conclusions

In this present work, a new hybrid membrane was developed using the complex modifier that consisted of equal amounts of heteroarm star macromolecules with a fullerene C_60_ core (HSM) and the [BMIM][Tf_2_N] ionic liquid (IL). The structural features of the hybrid PA/(HSM:IL) membrane as well as physical, mechanical, thermal, and gas transport properties were analyzed and compared with those of pristine PA and PA/HSM membranes. Capillary viscometry revealed the presence of macromolecular aggregates in the casting solutions; apparently, PA macromolecules become more compact after the addition of the complex modifier due to intermolecular interactions between membrane components in solution. SEM images of the membrane cross-sections demonstrated the cellular structure of the hybrid membrane and the presence of spherical domains uniformly distributed in the PA matrix.

Gas transport properties were studied by measuring He, O_2_, N_2_, and CO_2_ permeation through the pristine PA and hybrid membranes containing a 5 wt% modifier. The permeability coefficients of all gases depend on their effective molecular diameter and decrease after the inclusion of the complex modifier. At the same time, the ideal selectivity in the separation of He/N_2_, CO_2_/N_2_, and O_2_/N_2_ gas pairs increases for the hybrid membrane. The position of the PA/(HSM:IL) membrane on the Robeson’s diagram containing the data on O_2_/N_2_ selectivity for a variety of polymers is improved with respect to the position of the pristine PA membrane. The improvement in the transport properties of hybrid membranes is explained by the formation of supramolecular structures and domain morphology due to the ability of HSM arms to segregate. The (HMS:IL) complex modifier promotes a change of the membrane internal structure and redistribution of the free volume in the hybrid membrane.

The developed hybrid membrane exhibits good thermal stability and can be used in a wide temperature range. Mechanical tests show that the hybrid membrane has high values of strength and elongation at break; thus, these polymers will possess easy processability necessary for many applications.

## Figures and Tables

**Table 1:** Effective molecular kinetic diameters of gas molecules.

Gas	He	CO_2_	O_2_	N_2_
Diameter of gas molecule **(Å)**	1.8	3.01	2.83	3.0

**Table 2:** Some physical properties of polymer materials for membranes, 20°C.

Sample	[η], dL/g	T_g_, °C	Density, g/cm^3^
PA	2.66	260	1.326
PA/HSM	2.60	256	1.313
PA/(HSM:IL)	2.50	253	1.288

**Table 3:** Physical properties of membranes, 20°C.

Sample	Tensile Strength, MPa	Yield Strength, MPa	Elastic Modulus, MPa	Elongation at Break, %
PA	44	46	1700	70
PA/HSM	49	55	1850	50
PA/(HSM:IL)	45	53	1800	55

## Data Availability

The data presented in this study are available on request from the corresponding author.
